# 

**Published:** 1849-09

**Authors:** 


No. IV. SEPTEMBER. Vol. V.

### BUFFALO MEDICAL JOURNAL

AND

### MONTHLY REVIEW

OF

### MEDICAL AND SURGICAL SCIENCE.

EDITED BY AUSTIN FLINT, M. D.

BUFFALO:
PUBLISHED BY JEWETT, THOMAS & CO.
Commercial Advertiser Buildings.
1849.

